# Therapeutic Benefit of Extended Thymosin *β*4 Treatment Is Independent of Blood Glucose Level in Mice with Diabetic Peripheral Neuropathy

**DOI:** 10.1155/2015/173656

**Published:** 2015-04-07

**Authors:** Lei Wang, Michael Chopp, Longfei Jia, Xuerong Lu, Alexandra Szalad, Yi Zhang, RuiLan Zhang, Zheng Gang Zhang

**Affiliations:** ^1^Department of Neurology, Henry Ford Hospital, 2799 W. Grand Boulevard, Detroit, MI 48202, USA; ^2^Department of Physics, Oakland University, Rochester, MI 48309, USA

## Abstract

Peripheral neuropathy is a chronic complication of diabetes mellitus. To investigated the efficacy and safety of the extended treatment of diabetic peripheral neuropathy with thymosin *β*4 (T*β*4), male diabetic mice (db/db) at the age of 24 weeks were treated with T*β*4 or saline for 16 consecutive weeks. Treatment of diabetic mice with T*β*4 significantly improved motor (MCV) and sensory (SCV) conduction velocity in the sciatic nerve and the thermal and mechanical latency. However, T*β*4 treatment did not significantly alter blood glucose levels. Treatment with T*β*4 significantly increased intraepidermal nerve fiber density. Furthermore, T*β*4 counteracted the diabetes-induced axon diameter and myelin thickness reductions and the *g*-ratio increase in sciatic nerve. In vitro, compared with dorsal root ganglia (DRG) neurons derived from nondiabetic mice, DRG neurons derived from diabetic mice exhibited significantly decreased neurite outgrowth, whereas T*β*4 promoted neurite growth in these diabetic DRG neurons. Blockage of the Ang1/Tie2 signaling pathway with a neutralized antibody against Tie2 abolished T*β*4-increased neurite outgrowth. Our data demonstrate that extended T*β*4 treatment ameliorates diabetic-induced axonal degeneration and demyelination, which likely contribute to therapeutic effect of T*β*4 on diabetic neuropathy. The Ang1/Tie2 pathway may mediate T*β*4-induced axonal remodeling.

## 1. Introduction

Diabetes affects an estimated 346 million people worldwide [1]. Peripheral neuropathy is a long-term complication of diabetes mellitus which is associated with neurotrophic changes, degeneration, and demyelination of peripheral nerves [2, 3]. There is currently no effective treatment for preventing the development or reversing the progression of diabetic neuropathy. Thus, it is imperative to develop therapies for diabetic peripheral neuropathy.

Thymosin *β*4 (T*β*4), a major intracellular G-actin-sequestering 43-amino acid peptide, has multiple biological functions [4]. T*β*4 promotes axonal regeneration and remyelination as well as vasculogenesis [5, 6]. Preclinical studies have found that treatment with T*β*4 improves neurological function outcome after central and peripheral nervous system damage [5–7]. We previously demonstrated the fact that T*β*4 remarkably improved sciatic nerve vascular function and peripheral nerve function in a model of diabetic peripheral neuropathy [6]. However, the extended therapeutic effect of T*β*4 on axonal remodeling has not been investigated.

The angiopoietins (Ang), a family of endothelial cell growth factors, regulate vessel angiogenesis and stabilization [8, 9]. Ang1 also promotes neurite outgrowth in cultured dorsal root ganglion neurons and neuronal differentiation in neural progenitor cells [10, 11]. Overexpression of Ang1 in the brain alters neuronal dendrite configuration [12]. T*β*4 treatment of diabetic peripheral neuropathy reverses diabetes-reduced Ang1 expression in the sciatic nerve and thereby promotes vascular remodeling [6].

Diabetic peripheral neuropathy is a chronic disease. In the present study, we investigated the efficacy and safety of T*β*4 for the extended treatment of diabetic peripheral neuropathy. We found that extended T*β*4 treatment ameliorates diabetic-induced intraepidermal nerve fiber and sciatic nerve impairment, which likely contributes to functional recovery of diabetic neuropathy. In addition, our data indicate that the Ang1/Tie2 pathway may mediate T*β*4-induced axonal regeneration and remyelination in diabetic neuropathy. Thus, extended T*β*4 treatment may represent a safe and effective therapeutic approach for experimental diabetic neuropathy.

## 2. Material and Methods

### 2.1. Animals

All experimental procedures were carried out in accordance with the NIH Guide for the Care and Use of Laboratory Animals and were approved by the institutional Animal Care and Use Committee of Henry Ford Hospital. Male BKS.Cg-*m* ^+/+^ *Lepr* ^*db*^ */J* (db/db) mice (Jackson Laboratories, USA) aged 24 weeks were used. Age-matched heterozygote mice (db/m), a nonpenetrant genotype (Jackson Laboratories), were used as the control animals.

### 2.2. T*β*4 Treatment

db/db mice at age of 24 weeks were treated with T*β*4 at a dose of 30 mg/kg (RegeneRx Inc., USA, intraperitoneal injection, i.p.), daily for 16 weeks (*n* = 15/group). db/db mice (*n* = 15/group) of the same age were treated with same volume of saline and were used as a control group. Age-matched db/m mice, treated with saline (*n* = 15/group), were used as additional control groups. All mice were sacrificed 16 weeks after onset of treatment. Doses of T*β*4 were selected based on published studies [7].

Blood glucose levels were measured from the mouse tail vein by using an instant check meter (Roche Diagnostics, USA). Electrophysiological measurements, functional tests, blood glucose levels, and body weight were performed before treatment and then every 4 weeks until sacrifice. All procedures and analyses were performed by investigators who were blinded to the experimental group.

### 2.3. Neurophysiological Measurements

Sciatic nerve conduction velocity was assessed with orthodromic recording techniques, as previously described [13–15]. Briefly, mice were anesthetized with ketamine/xylazine (i.p., 100/10 mg/kg). The stimulating electrodes were plated at the knee and sciatic notch. Triggered single square wave current pulses were delivered using an isolated pulse stimulator (Model 2100, A-M Systems, USA). The simultaneous electromyographies were recorded by two sterilized electrodes placed in the dorsum of the foot with a Grass Amplifier (Model P5, Grass Instruments, USA). During the measurements, animal rectal temperature was kept at 37 ± 1.0°C using a feedback controlled water bath. Motor nerve conduction velocity (MCV) and sensory nerve conduction velocity (SCV) were calculated according to a published study [15].

### 2.4. Measurement of Thermal Sensitivity

To examine the sensitivity to noxious heat, plantar test was measured using a thermal stimulation meter (IITC model 336 TG combination tail-flick and paw analgesia meter; IITC Life Science, USA) according to published methods [16]. Briefly, mice were placed within a plexiglass chamber on a transparent glass surface and allowed to acclimate for at least 20 min. The meter was activated after placing the stimulator directly beneath the plantar surface of the hind paw. The paw-withdrawal latency in response to the radiant heat (15% intensity, cut-off time 30 sec) was recorded. At least five readings per animal were taken at 15 min intervals, and the average was calculated [6].

### 2.5. Tactile Allodynia Test

To examine tactile allodynia, we employed Von Frey filaments (Stoelting, USA) to stimulate paw withdrawal according to published protocols [17, 18]. Briefly, a series of filaments with force that ranged from 0.4 to 6.0 g were applied to the plantar surface of the left hindpaw with pressure causing the filament to buckle. A paw withdrawal in response to each stimulus was recorded and a 50% paw withdrawal threshold was calculated according to a published formula [17, 18].

### 2.6. Staining Myelin Sheets

The sciatic nerves were harvested at the mid-thigh level and fixed in the 2.5% glutaraldehyde and 0.5% sucrose (Sigma, USA) on PBS buffer for 6–8 hours and then immersed in 2% osmium tetroxide (Sigma) for 2 hours. The specimens were then dehydrated with numerous alcohol passages and embedded in paraffin [19]. Semithin transverse sections (2-*μ*m thick) were cut and stained with 1% toluidine blue and three semithin sections per mouse were analyzed.

### 2.7. Immunohistochemistry

The sciatic nerves were fixed in 4% paraformaldehyde for immunohistochemistry and then embedded in paraffin according to a published protocol [13]. Three cross sections (6-*μ*m-thick) or three longitudinal sections (6-*μ*m-thick) at 60 *μ*m apart per animal were used [13].

Epidermal foot pads from left hind feet were fixed in Zamboni's fixative for 2 hours, washed in PBS, and then kept in 30% sucrose/PBS overnight at 4°C. The samples were embedded in OCT compound and stored at −80°C. Three longitudinal 20-*μ*m-thick footpad sections from each mouse were prepared.

The following primary antibodies were used: polyclonal rabbit anti-myelin basic protein (MBP, 1 : 400, Dako Denmark, USA), polyclonal antineurofilament, heavy chain (NF-H, 1 : 1000, Thermo Scientific, USA), polyclonal rabbit anti-Ang1 (1 : 2000; Abcam, USA), polyclonal rabbit anti-S100 (1 : 400, Abcam, USA), and polyclonal rabbit anti-protein gene product 9.5 (PGP 9.5, 1 : 1,000; MILLIPORE, USA). Rabbit or goat IgG was used as a negative control. Sections were counterstained with 4′,6-diamidino-2-phenylindole (DAPI) (1 : 5000, Thermo Scientific, USA).

### 2.8. Image Acquisition and Quantification

Image analysis was performed using a computer imaging analysis system (MicroComputer Imaging Device, MCID, Imaging Research Inc., UK) [20].

For morphometric analysis of sciatic nerves, three sections spaced as 60 *μ*m interval for each staining were used for analysis from each mouse, and three fields of the view per section were randomly imaged under a 100x oil immersion objective (BX40; Olympus Optical, Japan). Myelinated fiber diameter, axon diameter, and myelin sheath thickness were measured. The *g*-ratio (the quotient axon diameter/fiber diameter) was calculated to measure the degree of myelination. At least 200 myelinated fibers were measured per animal [13, 21].

Intraepidermal nerve fiber profiles were digitized under a 40x objective (Carl Zeiss Axiostar Plus Microscope, USA) via the MCID system. The number of nerve fibers crossing the dermal-epidermal junction was counted and the density of nerves is expressed as fibers/mm length of section [22]. Representative images of intraepidermal nerve fibers were obtained by a laser-scanning confocal microscope (Zeiss LSM 510 NLO, Carl Zeiss, Germany).

For quantization of MBP, NF-H, and Ang1 immunoreactive, cross sections or longitudinal sections were digitized. Data are presented as the percentage of immunoreactive area within the total imaged area.

All analysis was conducted with the examiner who was blinded to the identity of the samples being studied.

### 2.9. Cell Culture

A normal glucose medium (NG) was defined as a medium containing 5 mM glucose, while a high glucose medium (HG) was referred to as a medium containing 30 mM glucose, which was chosen to match glucose levels prevalent in uncontrolled diabetic patients [23]. These glucose concentrations have been used for the in vitro hyperglycemia experiments by others [24, 25].

To examine the effect of T*β*4 on Schwann cells, primary mouse Schwann cells (MSCs) were cultured according to the manufactures' instructions (ScienCell Research Laboratories, USA).

### 2.10. Conditioned Media

To collect conditioned medium from MSCs, 2.5 × 10^6^ cells were plated onto a 100-mm-diameter dish in 10 mL of defined medium. The cells were cultured under the normal glucose or high glucose conditions in the presence or absence of T*β*4 (100 ng/mL) for 48 hours. The supernatant (conditioned medium) was collected, concentrated 10 times using 10 kD centrifugal filters (Amicon Ultra-15; Nihon Millipore, USA), and frozen at −80°C until use.

### 2.11. Primary Culture of DRG Neurons and Evaluation of Neurite Outgrowth

DRG neurons were harvested from 18–20 weeks diabetic db/db mice and nondiabetic db/m mice. Cultures were prepared according to a previously described procedure [26, 27] with some modifications. Briefly, DRG neurons were removed stripped of meninges and dissociated by a combination of Ca^2+^- and Mg^2+^-free Hanks balance salt solution (HBSS) containing 0.125% trypsin and 0.1% collagenase–A digestion for 30 min, and then mechanically triturated for ~20 times. Isolated DRG neurons were seeded on glass coverslips coated with laminin and plated at a density of 2,000 cells/well in a 24 well-plate in Neurobasal-A medium (Invitrogen, USA), 2% B-27 (GIBCO, USA), 2 mM GlutaMax, and 1% antibiotic-antimycotic. To evaluate the direct effect of T*β*4 on neurite outgrowth of DRG neurons, DRG neurons were cultured in DRG culture medium with or without T*β*4 (100 ng/mL) under normal glucose and high glucose condition. To evaluate the effects of conditional medium harvested from T*β*4 activated Schwann cells on neurite outgrowth, DRG neurons were cultured in DRG culture medium with one-tenth of the conditioned medium. After a 3-day culture, DRG neurons were performed for NF-H with Cy3 for neurite outgrowth measurement. To trace the neurite outgrowth of fluorescently labeled neurons, the fluorescent photomicrographs were captured at 20x magnification with a digital camera. Neurite outgrowth was measured in 20 neurons per coverslip. The total neurite lengths of each positive neuron were measured using MCID analysis system [28]. The average length of neurite outgrowth was presented.

### 2.12. Schwann Cell Proliferation and Migration

To investigate the effect of T*β*4 on Schwann cell proliferation, Schwann cells were seeded in a 24-well dish at a density of 1 × 10^4^ cells/well and incubated in normal and high glucose with T*β*4 at concentration 100 ng/mL for 72 hours. BrdU (10 *μ*mol/L) was added 12 hours prior to termination of the experiments. The cells were fixed for BrdU immunostaining. The number of BrdU positive cells was counted in 5 fields of view under a 20x objective.

To examine the effect of T*β*4 on migration of mouse Schwann cells, a modified Boyden's chamber assay was employed, as described previously [29]. Briefly, the polycarbonate filter (8 *μ*m pore size) (Neuro Probe Inc., USA) was coated with 50 *μ*g/mL fibronectin (Chemicon, USA) and 0.1% gelatin (Sigma) and placed between upper and lower chambers. The cells were preincubated in normal and high glucose levels with T*β*4 at concentration of 100 ng/mL in the presence or absence of the anti-Tie2 inhibitor (5 *μ*M) for 72 hours. Cell suspensions (5 × 10^4^ cells per well) were placed in the upper chamber, and the lower chamber was filled with medium containing human recombinant nerve growth factor (100 ng/mL, R&D Systems). The chamber was incubated for 5 hours at 37°C and 5% CO_2_. Migrating cells caught in the membrane were then stained using hematoxylin and eosin (Anatech LTD, USA). The number of cells that migrated through the filter was counted in 5 fields of view under a 40x objective.

### 2.13. Lactate Dehydrogenase (LDH) Cytotoxicity Assay

To determine cytotoxicity levels, the CytoTox 96 Non-Radioactive Cytotoxicity Assay kit (Promega, USA) was used following standard protocol. LDH levels were measured after 48-hour incubation period and LDH activity was detected by a plate reader at OD 490 nm. Data are presented as percentage of LDH level in the media to total LDH [9, 28].

### 2.14. Elisa Assay

To examine the effect of hyperglycemia on Ang1, Ang1 levels in supernatants were measured using an Elisa specific to detect mouse Ang1 according to the manufacturer's instructions (http://www.mybiosource.com/, USA).

### 2.15. Western Blot Analysis

Western blot was performed according to published methods [30]. Briefly, equal amounts of proteins were loaded on 10% SDS-polyacrylamide gel. After electrophoresis, the proteins were transferred to nitrocellulose membranes, and the blots were subsequently probed with the following antibodies: polyclonal rabbit anti-Ang1 (1 : 1000; Abcam). For detection, horseradish peroxidase-conjugated secondary antibodies were used (1 : 2000) followed by enhanced chemiluminescence development (Pierce, USA). Normalization of results was ensured by running parallel Western blot with *β*-actin antibody. The optical density was quantified using an image processing and analysis program (Scion Image, USA).

### 2.16. Statistical Analysis

For functional tests, data were evaluated for normality. Ranked data or nonparametric approach will be considered if the data are not normally distributed. The repeated measure analysis of variance (ANCOVA) was considered with dependent factor of time and independent factor of groups. The analysis started testing for group by time interaction, followed by the testing the main effect of group and subgroup analyses. Two-sample *t*-test or analysis of variance (ANOVA) was used to study the group difference on immunostaining, biochemistry, and Western blot, respectively. The data are presented as mean ± SE. A value of *P* < 0.05 was taken as significant.

## 3. Result

### 3.1. Extended T*β*4 Treatment Improves Neurological Functional Outcome in Diabetic db/db Mice

**Figure 1:**
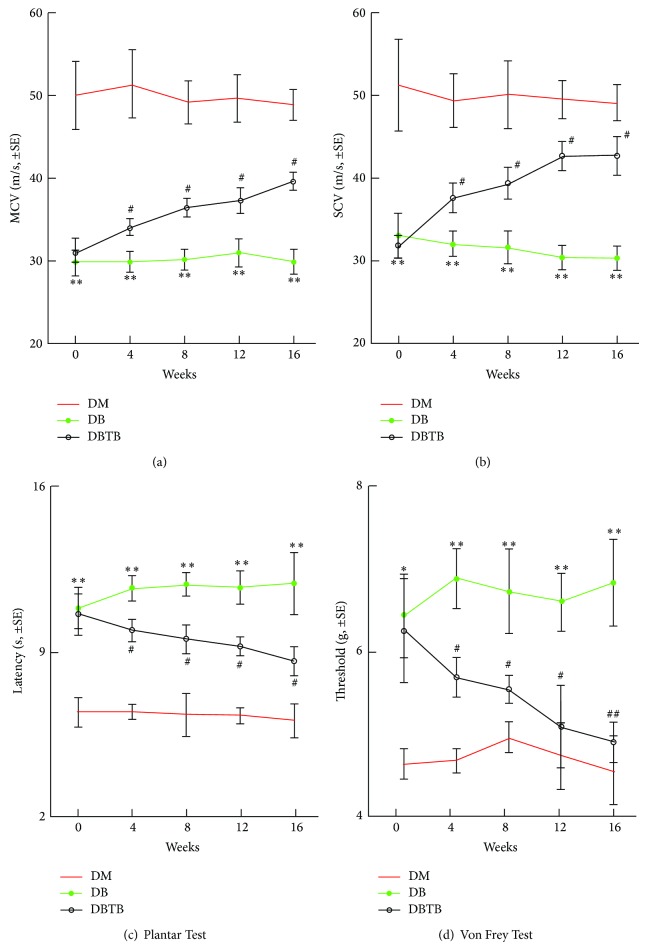
Effect of T*β*4 on neurological function in diabetic mice. Treatment of diabetic mice with T*β*4 improves neurological function measured by MCV (a), SCV (b), the Plantar test (c), and von Frey test (d). ^∗^ *P* < 0.05, ^∗∗^ *P* < 0.01 versus the nondiabetic group (DM). ^#^ *P* < 0.05 and ^##^ *P* < 0.01 versus the diabetic group (DB) treated with saline. *n* = 10/group.

Diabetic peripheral neuropathy is a chronic disease. We first test whether extended, that is, 16 weeks, T*β*4 treatment improves neurological function in diabetic neuropathy. Electrophysiological recording showed that treatment of db/db mice aged 24 weeks with T*β*4 (30 mg/kg) for 16 weeks significantly improved the MCV and SCV in the sciatic nerve compared with db/db mice treated with saline (Figures 1(a) and 1(b)). This increase in MCV and SCV started 4 weeks after treatment and persisted for 16 weeks during treatment, which was associated with substantial improvement in response to sensory function measured by the thermal and mechanical latency with plantar tests and tactile allodynia test, respectively (Figures 1(c) and 1(d)). Treatment with T*β*4 did not significantly alter blood glucose levels but increased animal body weight (Tables 1 and 2). These data indicate that extended T*β*4 treatment improves neurological outcomes without alteration of blood glucose levels.

### 3.2. Extended T*β*4 Treatment Promotes Axonal Regeneration and Remyelination in Peripheral Nerves

**Figure 2:**
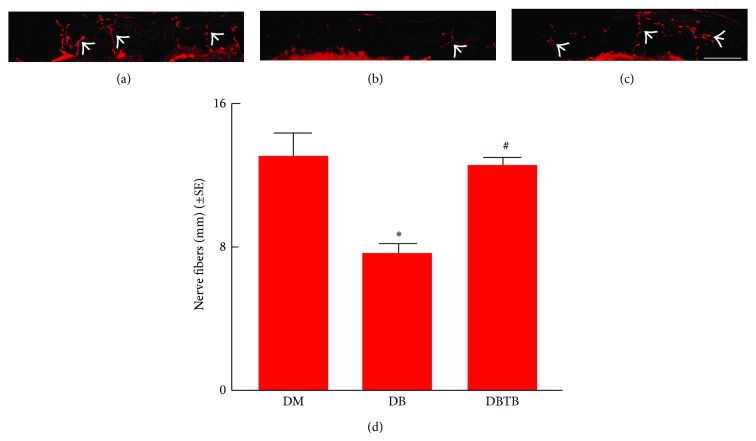
Effect of T*β*4 on the intraepidermal nerve fiber in diabetic mice. Panels (a) to (c) show PGP 9.5 immunoreactive fibers (arrows) in the plantar skin from a representative nondiabetic mouse (a), diabetic mouse treated with saline (b), and diabetic mouse treated with T*β*4 (c) 16 weeks after treatment. Panel (d) is quantitative data. Bar = 50 *μ*m. ^∗^ *P* < 0.05 versus the nondiabetic group (DM), ^#^ *P* < 0.05 versus diabetic mice treated with saline (DB). *n* = 10/group.

**Figure 3:**
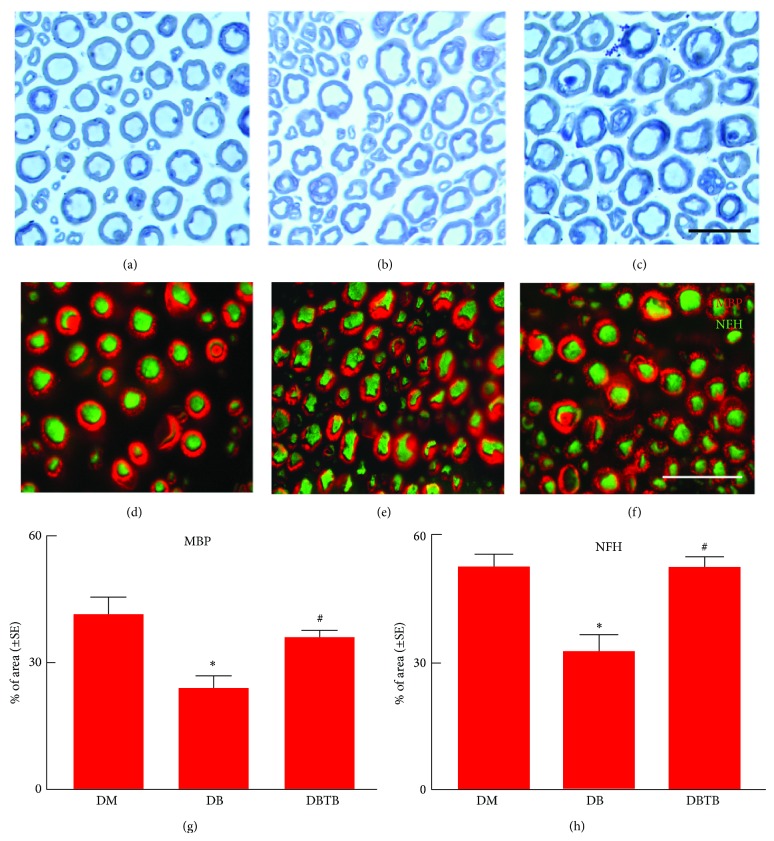
Effect of T*β*4 on MBP, NF-H, and morphometric change of myelinated sciatic nerves. Panels (a) to (c) show semithin toluidine blue-stained cross sections of sciatic nerves from a representative nondiabetic mouse (a), diabetic mouse treated with saline (b), and diabetic mouse treated with T*β*4 (c) for 16 weeks. Panels (d) to (f) are double immunofluorescent images showing MBP and NF-H immunoreactive sciatic nerves of a representative nondiabetic mouse (d), diabetic mouse treated with saline (e), and diabetic mouse treated with T*β*4 (f) for 16 weeks. Panels (g) and (h) show quantitative data of the percentage of MBP (g) and NF-H (h) immunoreactive area. Bars in *C* = 50 *μ*m and *F* = 25 *μ*m. ^∗^ *P* < 0.05 versus the nondiabetic group (DM) and ^#^ *P* < 0.05 versus diabetic mice treated with saline (DB). *n* = 10/group.

To examine whether the extended T*β*4 treatment affects distal nerve fibers, morphometric changes of nerve fibers were analyzed. Compared to nondiabetic db/m mice, diabetic db/db mice at age of 40 weeks exhibited substantial reduction of PGP 9.5 positive IENFs which is consistent with published studies showing loss of distal sensory nerve fibers in db/db mice and in patients with type II diabetes [31–33]. However, age-matched db/db mice treated with extended T*β*4 did not show significant reduction of IENFs compared to nondiabetic mice (Figure 2). Using toluidine-blue stained thin sections, we further analyzed morphometric changes of the sciatic nerve. Diabetic db/db mice treated with saline showed significant reduction in sciatic nerve fiber diameter and myelin sheath thickness and a significant increase in *g*-ratio (axon diameter/fiber diameter). In contrast, extended T*β*4 treatment completely reversed the sciatic nerve morphology changed by diabetes to levels comparable to nondiabetic db/m mice (Table 3 and Figures 3(a) to 3(c)). Moreover, double immunofluorescent staining showed that extended T*β*4 treatment significantly increased the number of NF-H positive sciatic nerves and MBP positive myelination (Figures 3(d) to 3(h)). Collectively, these data suggest that extended T*β*4 treatment enhances myelinated sciatic nerves and IENFs in diabetic mice.

### 3.3. T*β*4 Increases DRG Neuron Neurite Outgrowth and Schwann Cell Proliferation and Migration

**Figure 4:**
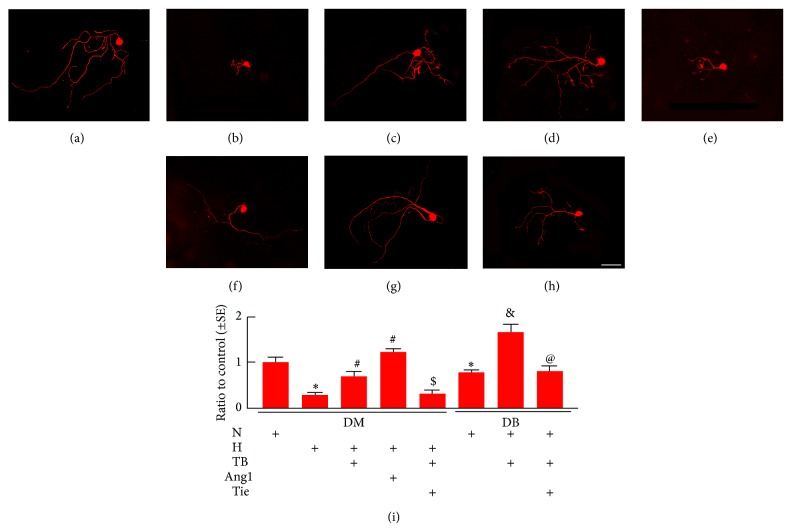
Effect of T*β*4 on DRG neurons neurite outgrowth in vitro. Panels (a) to (e) show NF-H immunoreactive nondiabetic DRG neuron cultured in normal glucose (N, (a)), high glucose (H, (b)), high glucose with T*β*4 (+TB, (c)), high glucose with Ang1 (+Ang, 100 ng/mL, (d)), and T*β*4 with antibody against Tie2 (+Tie, 5 *μ*g/mL, (e)). Panels (f) to (h) show NF-H immunoreactive diabetic DRG neuron cultured in normal glucose (f), normal glucose with T*β*4 (g), and T*β*4 with antibody against Tie2 (h). Panel (i) shows quantitative data of neurite outgrowth. Bar in *H* = 50 *μ*m. ^∗^ *P* < 0.05 versus the nondiabetic DRG neurons (DM) in normal glucose and ^#^ *P* < 0.05 versus nondiabetic DRG neurons in the high glucose. ^$^ *P* < 0.05 versus nondiabetic DRG neurons treated with T*β*4 in high glucose, ^&^ *P* < 0.05 versus diabetic DRG neurons (DB) in normal glucose, and  ^@^ *P* < 0.05 versus diabetic DRG neurons treated with T*β*4. *n* = 6/group.

**Figure 5:**
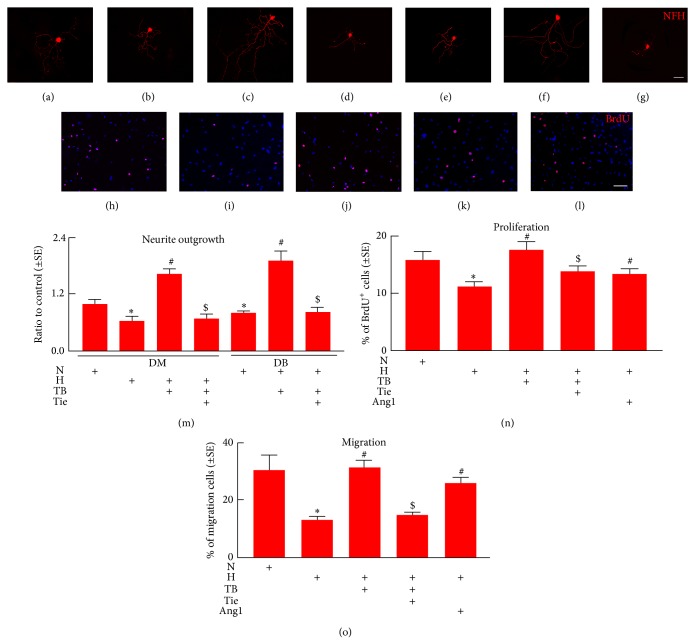
Effect of T*β*4 on DRG neurite outgrowth and Schwann cell proliferation and migration. Panels (a) to (d) show NF-H immunoreactive nondiabetic DRG neurons cultured in conditioned medium harvested from Schwann cells cultured with normal glucose (N, (a)), high glucose (H, (b)), high glucose with T*β*4 (+TB, 100 ng/mL, (c)), and T*β*4 with antibody against Tie2 (+Tie, 5 *μ*g/mL, (d)). Panels (e) to (g) show NF-H immunoreactive diabetic DRG neurons cultured in conditioned medium harvested from Schwann cells cultured with normal glucose (N, (e)), normal glucose with T*β*4 (TB, (f)), and T*β*4 with antibody against Tie2 (+Tie, (g)). Panels (h) to (l) show BrdU immunoreactive Schwann cells cultured in normal glucose (h), high glucose (i), high glucose with T*β*4 (j), T*β*4 with anti-Tie2 (k), and high glucose with Ang1 (l). Panel (m) shows quantitative data of neurite outgrowth from DRG neurons. Panel (n) shows quantitative data of the percentage of BrdU immunoreactive Schwann cells. Panel (o) shows quantitative data of migration cells assayed by a modified Boyden chamber. Bar in *G* = 50 *μ*m. *L* = 100 *μ*m. ^∗^ *P* < 0.05 and ^#^ *P* < 0.05 versus the normal glucose (N) and high glucose (H) and ^$^ *P* < 0.05 versus high glucose or normal glucose with T*β*4 group, respectively. *n* = 6/group.

To investigate the direct effect of T*β*4 on DRG neurons, a widely used in vitro model of primary DRG neurons was employed [26]. Under a regular glucose condition (5 mM glucose) which is comparable to a normal plasma glucose level measured in vivo, DRG neurons harvested from diabetic db/db mice at age of 18–20 weeks exhibited considerable reduction of neurite outgrowth compared to age-matched DRG neurons from nondiabetic db/m mice, whereas addition of T*β*4 into DRG neurons from db/db mice significantly promoted neurite growth (Figure 4). These data suggest that diabetic DRG neurons are not able to grow their fibers even under a normal glucose condition, while T*β*4 can promote neurite growth of these diabetic DRG neurons. We then cultured DRG neurons harvested from nondiabetic mice under a high glucose condition (30 mM) and found that high glucose conditions blocked neurite growth, whereas T*β*4 suppressed high glucose-induced inhibitory effect on neurite outgrowth (Figure 4). These data further support that T*β*4 can enhance neurite growth of DRG neurons under hyperglycemia conditions. Reduction of Schwann cells affects myelination [34]. We thus examined whether high glucose affects Schwann cell survival and proliferation. High glucose did not significantly affect Schwann cell survival measured by LDH assay (0.96 ± 0.01 versus 1 ± 0.01 in NG). However, high glucose significantly decreased the number of BrdU positive Schwann cells (Figures 5(h), 5(i), 5(j), 5(k), 5(l), and 5(n)). Moreover, a Boyden chamber assay revealed that high glucose decreased Schwann cell migration (Figure 5(o)). However, addition of T*β*4 in the presence of high glucose significantly attenuated the inhibitory effect of the high glucose on Schwann cell proliferation and migration (Figures 5(i), 5(j), 5(n), and 5(o)). These data suggest that, in addition to DRG neurons, T*β*4 promotes Schwann cell proliferation and migration under hyperglycemia condition.

### 3.4. T*β*4 Activated Schwann Cells Promote DRG Neuron Neurite Outgrowth

Schwann cells provide a microenvironment favoring axonal regeneration in the peripheral nervous system [35]. We then investigated whether T*β*4 activated Schwann cells secrete soluble factors that consequently improve DRG neurite outgrowth. DRG neurons harvested from nondiabetic db/m mice or diabetic db/db mice were incubated with conditioned media collected from Schwann cells that were cultured under regular and high glucose conditions with or without T*β*4. Compared to the conditioned medium collected from Schwann cells cultured under regular glucose condition, the conditioned medium derived from Schwann cells cultured under high glucose condition resulted in significant suppression of neurite outgrowth of DRG neurons derived from nondiabetic mice. In contrast, the conditioned medium collected from Schwann cells treated with T*β*4 under high glucose condition promoted neurite outgrowth in nondiabetic DRG neurons (Figures 5(a), 5(b), 5(c), and 5(m)). Moreover, the conditioned medium collected from Schwann cells treated with T*β*4 under normal glucose condition promoted neurite outgrowth in diabetic DRG neurons (Figures 5(e), 5(f), and 5(m)). These data suggest that soluble factors secreted by Schwann cells interact with DRG neurons in mediate neurite outgrowth.

**Figure 6:**
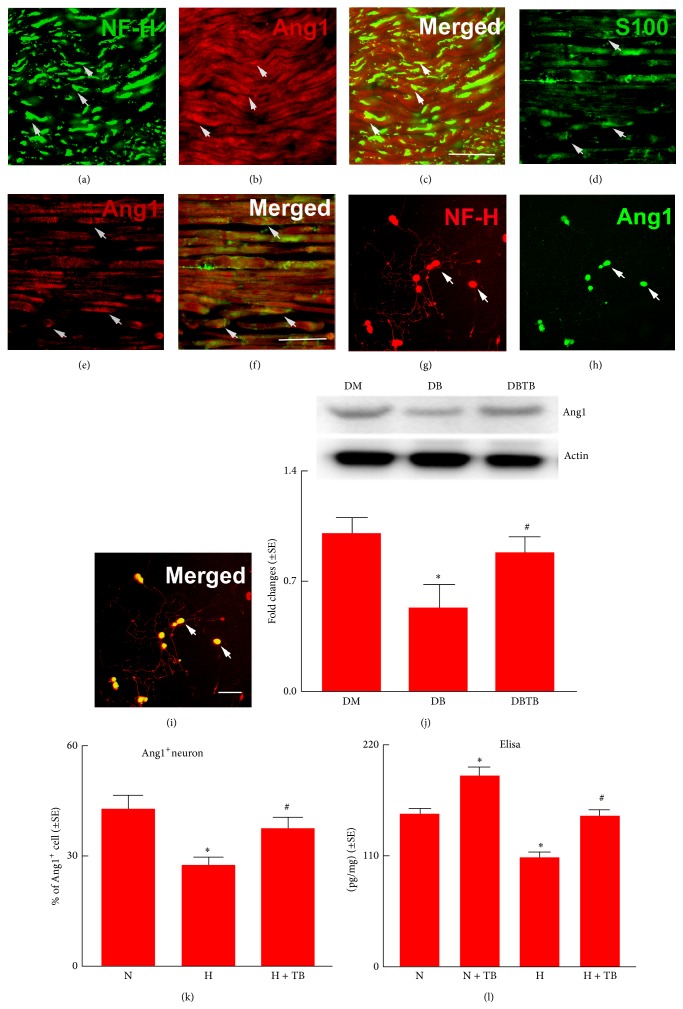
Effect of T*β*4 on Ang1 expression. Representative images of double immunofluorescent staining show that Ang1 immunoreactivity ((b), (c), (e), and (f) red, and arrows) was colocalized to NF-H positive axons ((a) and (c) green and arrows) and S100 positive Schwann cells ((d) and (f) green and arrows) in sciatic nerve tissue on the longitudinal section. Panels (g) to (i) show that Ang1 immunoreactivity ((h) and (i) green and arrows) was colocalized to NF-H positive cells ((g) and (i) red and arrows) in the cultured DRG neurons. Panel (j) shows Western blot analysis (j) of Ang1 in sciatic nerve tissue and *β*-actin was used as an internal control. Panel (k) shows quantitative data of the percentage of Ang1 immunoreactive DRG neurons cultured in normal glucose (N), high glucose (H), and high glucose with T*β*4 (+T*Β*, 100 ng/mL). Panel (l) shows Elisa data of Ang1 levels in supernatants harvested from Schwann cells in normal glucose (N), normal glucose with T*β*4 (N + TB), high glucose (H), and high glucose with T*β*4 (H + T*Β*). Bars in *C* and *F* = 50 *μ*m and *I* = 100 *μ*m. ^∗^ *P* < 0.05 versus the nondiabetic (DM) or normal glucose (N), ^#^ *P* < 0.05 versus diabetic mice treated with saline (DB) or high glucose (H), respectively. *n* = 6/group.

We previously demonstrated that a proangiogenic protein, Ang1, plays an important role in mediating development of diabetic sciatic nerve damage [6]. We thus examined whether Ang1 is involved in interaction between Schwann cells and DRG neurons to develop diabetic neuropathy. Consistent with our previous findings, we found that T*β*4 treatment abolished diabetic-reduced Ang1 expression in the diabetic sciatic nerve, measured by Western blot (Figure 6(j)). Double immunofluorescent staining showed that NF-H positive sciatic nerve and S100 positive Schwann cells were Ang1 positive (Figures 6(a) to 6(f)). Western blot analysis revealed that Ang1 protein was substantially decreased in diabetic sciatic nerve tissue compared to nondiabetic one (Figure 6(j)). To further examine whether glucose levels affect Ang1 expression in DRG neurons and Schwann cells, DRG neurons harvested from nondiabetic db/m mice and Schwann cells were cultured under normal and high glucose conditions. Immunocytochemistry showed that DRG neurons were Ang1 positive under regular glucose condition and that the high glucose substantially decreased Ang1 positive DRG neurons (Figures 6(g), 6(h), 6(i), and 6(k)). Incubation of DRG neurons with T*β*4 under high glucose condition significantly increased Ang1 positive DRG neurons (Figure 6(k)). Moreover, Elisa showed substantial reduction of Ang1 protein in supernatants harvested from Schwann cells cultured under high glucose condition compared to the supernatants collected from Schwann cells under regular glucose condition (Figure 6(l)), while T*β*4 reversed the effect of the high glucose on reduction of Ang1 protein (Figure 6(l)). These data indicate that high glucose downregulates Ang1 expression in DRG neurons and Schwann cells, which can be reversed by T*β*4.

To examine the cause effect of Ang1 on DRG neurons and Schwann cells, DRG neurons and Schwann cells were treated with Ang1 (100 ng/mL). Ang1 significantly increased DRG neurite outgrowth (Figures 4(d) and 4(i)) and Schwann cell proliferation (Figures 5(l) and 5(n)) and migration when cultured with the high glucose (Figure 5(o)). Using the neutralizing antibody against Tie2, we then blocked the Ang/Tie2 pathway in the presence of T*β*4. The antibody suppressed T*β*4 promoted neurite outgrowth of DRG neurons (Figures 4(e), 4(h), and 4(i); Figures 5(d), 5(g) and 5(m)) and Schwann cell proliferation (Figures 5(k) and 5(n)) and migration (Figure 5(o)) under high glucose condition. These data suggest that Ang1 mediates the effect of diabetes and T*β*4 on biological function of DRG neurons and Schwann cells.

## 4. Discussion

In this study, we demonstrate that extended T*β*4 treatment of diabetic mice improves neurological function of diabetic neuropathy, and the improvement is closely associated with amelioration of sciatic nerve axonal and myelin damage and an increase of intraepidermal nerve fiber density. In vitro experiments indicate that the Ang1/Tie2 signaling pathway likely mediates the effect of T*β*4 on axonal regeneration and remyelination.

Diabetic peripheral neuropathy is a chronic disease [27]. The goal of the current study was to assess the efficacy and safety of extended T*β*4 treatment on diabetic peripheral neuropathy. We found that administration of T*β*4 at 30 mg/kg for 16 consecutive weeks starting at animal aged 24 weeks substantially increased intraepidermal nerve fiber density, which was associated with considerable improvement of responses to thermal and mechanical stimuli. Diabetic db/db mice develop impairment of sciatic nerve conduction velocity starting at 8–14 weeks of age, while morphometric changes of axonal and myelin damage occur after 20 weeks of diabetes, which resemble human diabetic peripheral neuropathy [36]. Retraction of intraepidermal axons contributes to distal loss of sensation observed in diabetic peripheral neuropathy [37]. Thus, our data indicate that extended T*β*4 treatment is effective by enhancing regeneration of distal epidermal axons.

The present study suggests that the effect of T*β*4 on amelioration of diabetic peripheral neuropathy is unlikely related to hyperglycemia because the extended T*β*4 treatment did not reduce glucose levels. Our in vitro data indicate that primary DRG neurons harvested from diabetic mice cannot reverse their biological functions even when these cells were cultured under a physiological glucose condition. However, T*β*4 could overcome the detrimental effect of hyperglycemia on DRG neurons and Schwann cells. Our findings are consistent with studies published by others, demonstrating dissociation between hyperglycemia and peripheral neuropathy [38]. Use of insulin to control glucose levels in the physiological range fails to ameliorate diabetic peripheral neuropathy [38].

In addition to distal epidermal axons, T*β*4 treatment reduced axonal and myelin damage of the sciatic nerve. Morphological analyses reveal that the reductions in sciatic nerve fiber diameter and myelin thickness and increases in *g*-ratio, which is a reliable ratio for assessing axonal myelination in diabetic mice, were markedly restored by extended T*β*4 treatment. Therapies targeting axonal remodeling have been shown to enhance recovery of neurological function in experimental diabetic neuropathy. Treatment of db/db mice with gangliosides improves axonal morphometry and nerve conduction velocity [36]. Based on the present data together with published studies, we speculate that extended treatment with T*β*4 could normalize the distal epidermal axons and the morphology of the sciatic nerve which in turn ameliorates nerve conduction velocity (NCV) and sensory function damaged by diabetes.

In the peripheral nervous system, Schwann cells regulate peripheral nerve remyelination and regeneration by their capacity to proliferate and migrate [39, 40]. Many neurotrophic factors regulate axonal remodeling. Ang1 is interesting because it not only promotes angiogenesis, but also acts as a neurotrophic factor on neurons [41]. The present study shows that hyperglycemia downregulates Ang1 expression in DRG neurons and Schwann cells, which contributes suppression of DRG neuron neurite outgrowth and Schwann cell proliferation and migration. T*β*4 reverses the effect of hyperglycemic-reduced Ang1 expression by facilitating neurite outgrowth in DRG neuron and proliferation and migration of Schwann cells. Thus, our data indicate that Ang1 mediates T*β*4-improved DRG neuron and Schwann cell biological function.

The regeneration of myelinated nerve fibers depends on interactions between Schwann cells and axons [38]. Schwann cells provide a microenvironment favoring axonal regeneration due to secretion of neurotrophic factors [42–44]. Hu et al. reported that Schwann cells promote neurite outgrowth of DRG neurons by secreting NGF [45]. The present study showed that Ang1 in conditioned medium from T*β*4-stimulated Schwann cells enhances DRG neurite outgrowth. Hence, Ang1 participates in the crosstalk between Schwann cells and axons during axonal remodeling. We previously demonstrated that T*β*4 upregulates Ang1 in blood vessels, which leads to restoration of vascular function in the diabetic sciatic nerve [6]. The present study showed that T*β*4 also upregulates Ang1 in DRG neurons and Schwann cells under hyperglycemia condition. Others have shown that Ang1 improves regeneration of nerve fibers in diabetic ob/ob mice [46]. Thus, activation of the Ang/Tie2 pathway by T*β*4 likely facilitates axonal regeneration and remyelination, leading to improvement of peripheral nerve function under diabetic neuropathy. However, other potential neurotrophic factors may also be involved in T*β*4-enhanced axonal remodeling, and further studies are warranted.

In summary, our results demonstrate that extended T*β*4 treatment is an effective and safe therapeutic approach to ameliorate experimental diabetic neuropathy. The Ang1/Tie2 signaling pathway likely plays a significant role in the therapeutic effect of T*β*4 on diabetic neuropathy.

## Figures and Tables

**Table 1:** Effect of T*β*4 on blood glucose.

Groups	Blood glucose (g/dL)				
	0 w	4 w	8 w	12 w	16 w
DM + saline	155 ± 4.19	167 ± 5.2	141.1 ± 4.3	136.8 ± 5.7	125.7 ± 5.4
DB + saline	524 ± 4.3^∗^	524 ± 17.4^∗^	574 ± 3.9^∗^	555 ± 14.6^∗^	533 ± 71.7^∗^
DB + TB4	523.3 ± 10.5^∗^	515 ± 13.2^∗^	565 ± 9.6^∗^	517 ± 22.8^∗^	531 ± 12.2^∗^

Values are mean ± SE. ^∗^ *P* < 0.01 versus DM + saline group. *n* = 10/group. W = week; 0 w represents before the treatment, while other numbers indicate after the treatment. DM = nondiabetic mouse; DB = diabetic mouse; TB4 = T*β*4.

**Table 2:** Effect of T*β*4 on body weight.

Groups	Weight, g				
	0 w	4 w	8 w	12 w	16 w
DM + saline	31.5 ± 0.3	32.2 ± 0.24	32.2 ± 0.24	33.0 ± 0.34	33.2 ± 0.26
DB + saline	56.4 ± 1.6^∗^	48.5 ± 0.87^∗^	43.4 ± 1.0^∗^	43.9 ± 1.01^∗^	39.8 ± 1.04^∗^
DB + TB4	58.0 ± 0.6	56.7 ± 0.91^#^	54.9 ± 1.18^#^	56.0 ± 1.49^#^	53.6 ± 1.7^#^

Values are mean ± SE. ^∗^ *P* < 0.01 versus DM + saline group. ^#^ *P* < 0.01 versus DB + saline group. *n* = 10/group. W = week; 0 w represents before the treatment, while other numbers indicate after the treatment. DM = nondiabetic mouse; DB = diabetic mouse; TB4 = T*β*4.

**Table 3:** Effect of T*β*4 on histomorphometric parameter of sciatic nerves.

Property	DM	DB	
	+ saline	+ saline	+ T*β*4
Fiber diameter (*µ*m)	8.59 ± 0.06	7.68 ± 0.08^∗∗^	8.6 ± 0.07^##^
Axon diameter (*µ*m)	5.04 ± 0.05	4.78 ± 0.06^∗∗^	4.98 ± 0.06^#^
Myelin thickness	1.78 ± 0.05	1.50 ± 0.04^∗^	1.85 ± 0.04^#^
*G* ratio	0.59 ± 0.008	0.61 ± 0.008^∗^	0.57 ± 0.008^#^

Values are mean ± SE. ^∗^ *P* < 0.05, ^∗∗^ *P* < 0.01 versus DM + saline group. ^#^ *P* < 0.05, ^##^ *P* < 0.01 versus DB + saline group. *n* = 10/group. W = week; 0 w represents before the treatment, while other numbers indicate after the treatment. DM = nondiabetic mouse; DB = diabetic mouse; TB4 = T*β*4.
